# End-of-Life Preferences in Older US Adults—Bridging the “What Matters” Chasm

**DOI:** 10.1001/jamanetworkopen.2021.42279

**Published:** 2022-01-04

**Authors:** Anand S. Iyer, Cynthia J. Brown

**Affiliations:** Division of Pulmonary, Allergy, and Critical Care Medicine, Department of Medicine, University of Alabama at Birmingham, Birmingham; Center for Palliative and Supportive Care, Division of Gerontology, Geriatrics, and Palliative Care, Department of Medicine, University of Alabama at Birmingham, Birmingham; Department of Medicine, Louisiana State University Health Sciences Center, New Orleans.

The first M of the 4Ms Framework for Age-Friendly Healthy Systems (what matters, medications, mentation, and mobility) refers to understanding the values of older adults and establishing their preferences for care across the continuum.^1^ For older US adults at the end of life, a “what matters” chasm has formed between the estimated 70% who express a preference to die at home and the 30% who actually do.^2^ This discordance could be attributable in part to the variable access to and underfunding of home and end-of-life care across the US. An increasing number of people growing older with serious illnesses and dying at home will need home and community-based services (HCBS) to help with activities of daily living and staying independent.^3^ However, the US health care system does not place enough emphasis on or provide sufficient funding for HCBS and is not ready for the seismic shifts toward more in-home deaths anticipated with the aging population during the next decade. Policy makers must allocate more resources to HCBS and increase access to early palliative care to ensure patients and their families have all they need in place for a peaceful death at home if that is desired.

In this issue of *JAMA Network Open*, Abe and colleagues^4^ present a multilevel, cross-sectional regression analysis of long-term care claims data from 544 836 older Japanese decedents to understand the contribution of individual, municipal, and prefectural (roughly akin to state-level) characteristics on the variation in place of death. A striking result was that despite the preference of 55% of older Japanese adults for an in-home death, only 10% of decedents in this cohort actually died at home. These decedents had a lower degree of care needs, were married, and had nonacute diseases, such as cancer or dementia. Municipalities with more clinics, physicians, and in-home care workers had more in-home deaths. Using mixed-effects logistic regression, Abe et al^4^ found that municipal-level characteristics were associated with 7% of the variance in place of death compared with 3% for prefecture-level characteristics, and municipal characteristics were significantly associated with in-home deaths.

Japan has universal health insurance, and although local budgets and taxes are controlled centrally, their system for provisions such as HCBS are administered at the local level by municipalities. This system is in contrast to that in the US, where HCBS are largely funded through Medicaid at the state level. Despite these differences, there are several lessons to learn. First, half of Medicaid spending in the US for long-term care goes to HCBS through Medicaid waivers. States voluntarily and variably participate in these services and cap enrollment, so HCBS waiting lists with nearly a million people on them continue to increase. Expanding Medicaid funding for HCBS could help; however, that is an uphill battle, and many states, especially those in the rural South, failed to expand Medicaid. Provisions within the American Rescue Plan provide a possible way to expand Medicaid and help older adults find access to quality HCBS and prepare for a peaceful end of life in these underresourced areas.^5^

Second, having access to more days of in-home care services is associated with more deaths at home; however, greater access will require more in-home care workers.^6^ Working as a home health aide is 1 of the fastest growing low-wage jobs in the US, but whether these jobs can be filled with a stagnant workforce, gross underpayment, and undervaluing of the profession is an important problem to solve. Most of the approximately 3 million in-home care workers are middle-aged Black women, and their mean annual earnings range from $13 000 to $27 000,^7^ which puts approximately one-quarter of in-home care workers under the federal poverty line. Increasing wages for this essential workforce may draw more to the profession and could bridge some of the “what matters” chasm regarding place of death.

Another important finding in the current analysis^4^ was that nearly half of decedents had spouses, which was associated with more in-home deaths. An estimated $500 billion of care in the US is provided by unpaid family members, who are a pillar of our medical system and yet receive little to no help or training. Many are spouses who are aging and have functional limitations or serious illnesses themselves, and nearly half do this without any help.^8^ Provision of daily care without training, respite, or reimbursement results in substantial burden. Our health care system has yet to devise a solution to support and compensate them appropriately.

Not being a burden, minimizing symptoms at the end of life, and maintaining basic functional status matter to older US adults.^2^ However, as further evidence of a widening “what matters” chasm, at least a quarter of older US adults with serious illnesses have difficulty getting help with activities of daily living, and a third of older adults and their families have unmet emotional or spiritual support needs.^9^ Increased HCBS access could reduce some of these burdens, and provisions within the Build Back Better Act could help. For example, the HCBS Improvement Program increases federal Medicaid matching funds for these services by 7%.^5^ Other provisions make permanent programs, such as Money Follows the Person, and impoverishment provisions for spouses, which have helped more than 100 000 older adults and people with disability move from institutional to community settings during the past decade.

Finally, our health care system has no solution for older adults who prefer to die at home but have a high degree of care needs or who live alone and do not have a care partner or documented surrogate decision-maker. These adults likely end up in the hospital at the end of life. In the current study, municipalities in Japan with more hospital beds had fewer in-home deaths, which also likely happens in the US, where the focus is generally placed on increasing access to acute care but not on supporting HCBS proportionately. As a result, people living with serious illnesses, such as chronic obstructive pulmonary disease, can experience a chaotic end of life with anxiety-provoking breathlessness and frequent hospitalizations. Earlier access to palliative care could help them proactively prepare for the challenges of managing distressing symptoms at home, improve the low rates of advance care planning, and facilitate documentation of “what matters” to them at the end of life. Policies such as the Palliative Care and Hospice Education and Training Act^10^ could also increase the cadre of people trained in primary palliative care who are capable of bridging the chasm from the frontlines of every specialty and helping prepare older adults for the end of life. Likewise, supporting hospice programs to provide around-the-clock nursing access or expanding the scope of practice of personal care aides to do more advanced and medical-focused tasks could reduce some of the burden experienced by patients and their families in their most trying times.

Bridging the “what matters” chasm is everyone’s responsibility. The data from the study by Abe et al^4^ suggest that improving access to HCBS could be 1 way to reduce the discordance between preferred and actual place of death for older adults. More funding and support for HCBS are needed so that older US adults living with serious illness and their families can make decisions about care near the end of life based on what is readily available to them at home rather than on what is not.
